# Primary diffuse large B-cell lymphoma of the fallopian tube treated with a combination of surgery and chemotherapy: Case report

**DOI:** 10.1097/MD.0000000000024049

**Published:** 2021-01-22

**Authors:** Ye Zhou, Chao Zhang, Yingying Gong, Linqing Yang, Yunfei Wang

**Affiliations:** a Department of Gynecology, Affiliated Hospital of Jining Medical University, Jining Medical University; b School of Clinical Medicine Jining Medical University, Shandong, China.

**Keywords:** diffuse large B-cell lymphoma, extranodal lymphoma, non-hodgkin lymphoma, primary fallopian tube lymphoma

## Abstract

**Rationale:**

Primary female genital tract lymphomas are sporadic neoplasms, accounting for 0.2% to 1.1% of all cases of extranodal lymphoma. The most common genital localizations are the cervix, the uterine corpus and the ovary, while primary lymphomas of the fallopian tube are quite unusual. According to literature searching in PubMed, this is the first reported case of primary diffuse large B-cell lymphoma of the fallopian tube.

**Patient concerns:**

A 52-year-old woman presented with a more than 2 months history of intermittent lower abdominal pain. The gynecological examination showed that the uterus, as big as 3 months of pregnancy, had weak activity and no tenderness. The uterine rectum lacuna was like a hard nodule of about 3 × 2 cm, and an irregular solid mass was fixed and inactive in the right adnexa.

**Diagnoses:**

In accordance with Ann Arbor staging system, a stage IE primary diffuse large B-cell lymphoma of fallopian tube was diagnosed for this patient, based on the tumor pathology, the results of bone marrow biopsy and computed tomography (CT) scan.

**Interventions:**

After gynecological/urinary ultrasound, blood test, pelvic contrast enhanced CT scan and CT angiography of iliac artery, exploratory laparoscopy and following hysterectomy with bilateral salpingo-oophorectomy were performed. After the surgery, the patient was treated with combined Rituximab and chemotherapy and got complete response (CR).

**Outcomes:**

After the operation and R-CHPOP, following up for more than 1 year so far, the patient has no tumor recurrence and is still in good condition.

**Lessons:**

It is very difficult to diagnose the primary diffuse large B-cell lymphoma of fallopian tube, not only because of its rarity, but also because of its non-specific clinical manifestations. It easily be treated as late ovarian cancer by gynecologist. So the pathology diagnosis and surgeons’ decision is very important. Because lymphoma is pretty sensitive to chemotherapy and easy to get complete response, so we no need to do an operation like ovarian cancer and should put chemotherapy as a primary method for lymphomas of the female genital tract.

## 1 Introduction

Primary gynecologic tract lymphoma accounts for 0.2% to 1.1% of all cases of extra-nodal lymphoma.^[1]^ The most prevalent tumor histological subtype was Diffuse large B-cell lymphoma (DLBCL), followed by follicular lymphoma, Burkitt lymphoma and Mucosal-associated lymphoid tissue lymphoma.^[2]^ The most common genital localizations are the cervix, the uterine corpus, the ovary and the vagina/vulva.^[2,3]^ The primary lymphomas of the fallopian tube are quite unusual. According to literature searching in PubMed, we only found case reports about follicular lymphoma, T-cell lymphoma and marginal zone B-cell lymphoma happened in the fallopian tube, but no DLBCL reported. In this article, we present a case with a primary extranodal DLBCL of the fallopian tube in a middle-aged woman, who was diagnosed based on histopathological and immunohistochemical findings. This case was first treated at the Department of Gynecology, Affiliated Hospital of Jining Medical University. The patient provided signed informed consent, and Institutional Review Board approval was obtained (No.2019C002).

## 2 Case report

**Figure 1:**
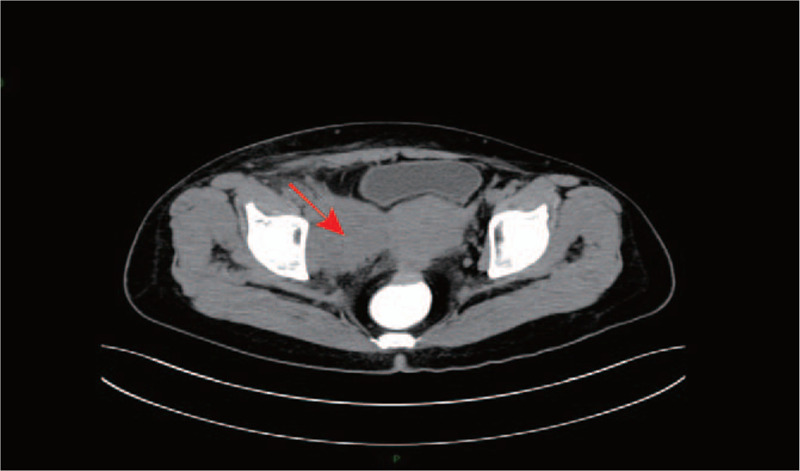
Plain and enhanced spiral CT scan of the pelvis showed soft tissue mass in the right adnexa, about 8.2 cm × 6.5 cm × 5.0 cm in size (shown by the red arrow).

A 52-year-old woman from China presented with a more than 2 months history of intermittent lower abdominal pain. She had no abdominal distension, fever or wasting, but accompanying with swelling in the right lower limb (The circumference of the right thigh was 46.5 cm, right lower leg was 38.3 cm, while left thigh was 43.2 cm and left lower leg was 37.3 cm). The skin temperature of the right lower extremity is higher than that of the left. The gynecological examination showed that the vulva, vagina, and cervix was regular, while the uterus, as big as 3 months of pregnancy, had poor activity and no tenderness. The uterine rectum lacuna was like a hard nodule of about 3 × 2 cm. At the same time, Pelvic examination revealed an irregular solid mass that was fixed and inactive of the right adnexa. No abnormal signs were found in the left adnexa. There was no swelling of superficial lymph nodes. No obvious abnormality was found in the blood routine, blood coagulation routine and liver function. The patient's previous medical history, clinical examination and other laboratory tests did not indicate any related autoimmune/immunocompromising disease or any other serious illness. Tumor markers: CA125 99 U/ml, HE4 110.7 pmol/L, CEA 1.84 ng/ml, AFP 3.95 ng/ml, CA199 <2.00 U/ML. Gynecological ultrasound examination showed that the right adnexal area had a cystic solid mass, about 4.9 cm × 3.9 cm × 3.7 cm in size, with clear boundary, poor sound transmission of cystic part, uneven echo of solid part, and accessible blood flow signal. Urinary ultrasound: the upper segment of the right ureter was dilated, about 1.0 cm width in a relatively wider place, and the hypoechoic mass was found at the iliac blood vessel, about 8.2 cm × 7.2 cm × 7.1 cm in size, with unclear boundary, irregular shape, uneven internal echo, and abundant arterial blood flow signals could be detected. Plain scan and enhanced scan of pelvic spiral computed tomography (CT): soft tissue mass is seen in the right adnexal area, about 8.2 cm × 6.5 cm × 5.0 cm in size (Fig. 1). Double source CT angiography of iliac artery showed that the focus surrounded the right common iliac artery and internal and external iliac vessels, the right ureteropelvic segment was involved, and the right renal pelvis and upper middle ureter were dilated, in hydronephrosis and delayed perfusion and excretion of the right kidney.

**Figure 2:**
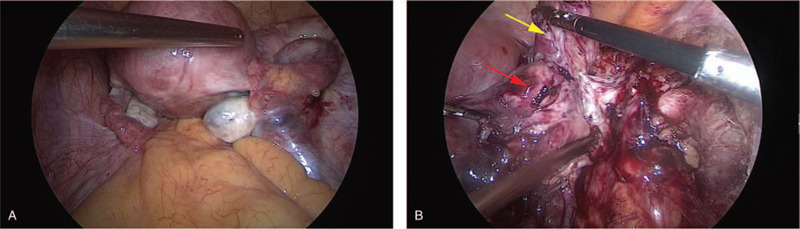
During the operation, the right fallopian tube was thickened and hard, about 7 x 6 x 3 cm in size, and the right round ligament was thickened and hard, about 3 cm in diameter, adhered to the right fallopian tube. The right ureter and the right iliac blood vessel were dilated (A). In the image B, right fallopian tube (as red arrow), right round ligament (as yellow arrow).

**Figure 3:**
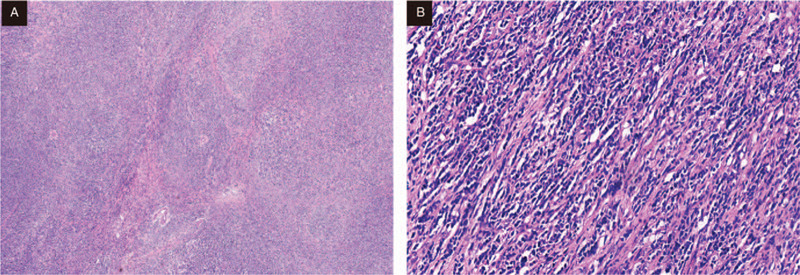
Sections from the right oviduct showing sheets of tumor cells high nucleocytoplasmic ratio, round to irregular nuclei, coarse chromatin, occasional prominent nucleoli, and scanty cytoplasm (H&E; A:4 × ; B:20 × ).

**Figure 4:**
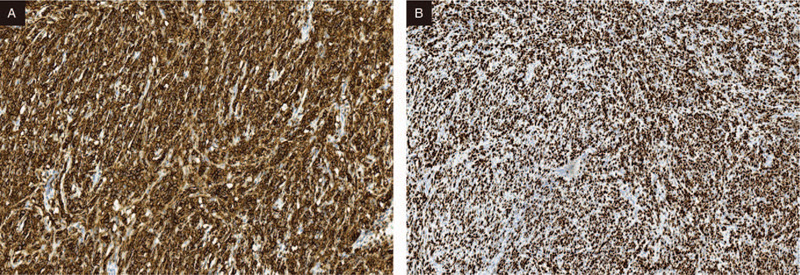
(A) Immunohistochemical staining for CD20 showing strong diffuse membranous positivity in the tumor cells (20 × ). (B) Immunohistochemical staining for Ki-67 showing high Ki-67 index (10 × ).

A laparoscopy was performed under the presumptive diagnosis of pelvic mass. During the operation, the right fallopian tube was thickened and hard, the size was about 7x6x3 cm, and the right round ligament was thickened and hard, the diameter was about 3 cm, which was adhered to the right fallopian tube, with the right ureter and the right iliac blood vessel dilated (Fig. 2). Hysterectomy-bilateral salpingo- oophorectomy was operated and delivered for rapid pathology. Gross images showing: the right fallopian tube was thickened, 7 × 6 × 3 cm in size, gray, hard, irregular in shape, part of the surface still smooth, dense adhesion with the right round ligament, unclear boundary. The size of the right ovary was 3 × 1.8 × 1.3 cm, and a cystic cavity with a diameter of 1 cm could be seen through incision, which was smooth; there was no obvious abnormality in the appearance of the left fallopian tube with a length of 8 cm, and the size of the left ovary was 3 × 1.5 × 1 cm. The pathological results showed that large numbers of hyperchromatic cells infiltrated and grew in the right round ligament and the right fallopian tube wall, the cells were the same, and the mitotic image was visible, which should be further confirmed by paraffin. Paraffin pathology after operation: (right adnexal area and round ligament) For diffuse large B-cell lymphoma (source of germinal center) (Fig. 3), the tumor size was 7 × 7 × 3 cm, and focal invasion of the myometrium and the whole layer of the fallopian tube wall was operated; there is no tumor at bilateral ovarian tissue; there was no tumor at left fallopian tube tissue with vasodilation and hyperemia. As expected, immunohistology demonstrated: CK (-), LCA (+), CD20 (+), CD3 (+), Ki-67 (+70–80%), CD10 (+), MPO (-), Bcl-6 (+), Mum-1(-) (Fig. 4).

**Figure 5:**
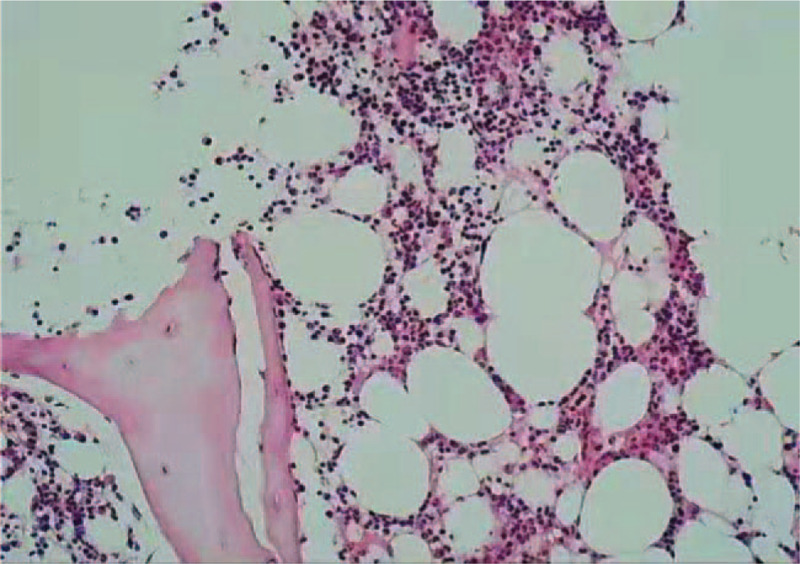
Bone marrow biopsy: the degree of nucleated cell proliferation in bone marrow is more active (65%), and the proportion of granulocytes/red cells is slightly reduced; granulocytes are mainly in the mature stage, while a few are in the immature stage; erythrocytes are mainly in the middle and late stage; megakaryocytes are mainly in the normal range, mainly the lobulated nucleus; lymphocytes are scattered, but no significant increase is found; no fibrosis is found in bone marrow stroma. (H&E;10 × ).

Following the diagnosis of lymphoma, bone marrow biopsy and cerebrospinal fluid analysis were performed. The results did not show disease involvement (Fig. 5). Then the patient chose another hospital for further therapy (department of hematology of Qilu hospital). In this hospital, neck, thorax, and abdomen CT scan was operated. No evidence of disease was found outside the pelvis, and she was classified according to the Ann Arbor staging system as stage IE (involvement of a single extralymphatic site or organ). She was treated with Rituximab injection + CHOP (6 cycles) and achieved CR. After following up for 1 year so far, the patient is still in good condition.

## 3 Discussion and conclusion

Primary non-Hodgkin's lymphoma (NHL) is mainly derived from lymph nodes or lymphangiocytes of extranodal organs.^[4]^ Its global incidence is about 3%, and the incidence of extranodal lymphoma accounts for 15% of NHL, whose clinical manifestations are diverse.^[5]^ Primary lymphoma of the female genital tract (PLFGT) is a very rare entity, comprising 0.2–1.1% of all cases of extra-nodal lymphoma. PLFGT most commonly can arise from the ovary, uterus, cervix, vagina and vulva. A lot of cases analysis studies of PLFGT confirmed the above conclusion.^[2,6–8]^ However, there was no report on the primary NHL of fallopian tube.

Primary lymphoma of the fallopian tube is extremely rare and most cases of fallopian tube involvement reflect more extensive disease arising in the ovary, with spread to the fallopian tube. Only rare cases of primary tubal lymphoma have been reported. However, all cases reported so far have not been DLBCL. These include 3 cases of primary extranodal marginal zone B-cell lymphoma (MALT-type lymphoma) of the fallopian tube,^[9]^ 4 cases of primary follicular lymphoma of the fallopian tube^[10,11]^ and one case of primary T-cell lymphoma of bilateral fallopian tubes.^[12]^

Here, we describe a new case of primary DLBCL arising in the fallopian tube, clinically presented with intermittent hypogastralgia, pelvic mass and compression symptoms. As NHL is rarely seen in female genital system, it can be easily confused with any other common gynecologic malignancy. Therefore, it's very difficult to do an early diagnosis in most patients and patients are often diagnosed by results obtained from intraoperative or postoperative pathological evaluations.^[13]^ Once the NHL has been diagnosed, it must be staged, as staging has proved to be 1 of the most important factors to predict survival. Clinical examinations and other investigations including CT scan, full blood count, renal and liver function test, bone marrow aspirate and so on must be performed to set the stage of the tumor, following the Ann Arbor staging classification for extranodal lymphomas. In this case, peripheral blood, bone marrow puncture and outside pelvic CT scan showed no obvious abnormality, no history of lymphoma. Before operation, pelvic CT and gynecological ultrasound showed the mass of adnexa. After bilateral adnexectomy of the whole uterus, pathology showed diffuse large B-cell lymphoma of the right fallopian tube (source of germinal center). The tumor foci invaded the myometrium and the wall of the fallopian tube; no tumor was found in bilateral ovarian tissues. Therefore, it is considered that the primary tumor is in the fallopian tube.

Based on this staging classification, the involvement of a single extra-lymphatic organ or site is defined as stage IE, as our patient. Due to the rarity of the disease, there is no standardized treatment plan at present. It is generally considered that this disease is a kind of malignant tumor sensitive to radiotherapy and chemotherapy. CHOP (cyclophosphamide, doxorubicin, vincristine and prednisolone) chemotherapy combined with rituximab was used in the initial and recurrent treatment of aggressive NHL, which improved the overall survival rate of DLBCL.^[14,15]^ Our patient was treated with a 6-cycles Rituximab and chemotherapy and achieved a complete response. Following up for 1 year, the patient is still in good condition.

In conclusion, it is very difficult to diagnose the primary diffuse large B-cell lymphoma of fallopian tube, not only because of its rarity, but also because of its non-specific clinical manifestations. This report firstly introduces a new rare case of diffuse large B-cell lymphoma in the fallopian tube, presenting the whole diagnosis and treatment process of the disease, followed up closely and reported continuously.

## Author contributions

**Conceptualization**: Ye Zhou, Linqing Yang, Yunfei Wang

**Data curation**: Ye Zhou, Chao Zhang, Yingying Gong

**Investigation**: Chao Zhang, Yunfei Wang

**Methodology**: Ye Zhou, Yunfei Wang

**Project administration**: Yunfei Wang

**Supervision**: Yunfei Wang

**Writing – original draft**: Ye Zhou

**Writing – review & editing**: Linqing Yang, Yunfei Wang
